# Economic Evaluation of Pharmacogenetic Tests in Patients Subjected to Renal Transplantation: A Review of Literature

**DOI:** 10.3389/fpubh.2016.00189

**Published:** 2016-08-31

**Authors:** Nemanja Rancic, Viktorija Dragojevic-Simic, Neven Vavic, Aleksandra Kovacevic, Zoran Segrt, Natasa Djordjevic

**Affiliations:** ^1^Centre for Clinical Pharmacology, Military Medical Academy Medical Faculty, University of Defence, Belgrade, Serbia; ^2^Solid Organ Transplantation Center, Military Medical Academy, Belgrade, Serbia; ^3^Management of the Military Medical Academy, Military Medical Academy Medical Faculty, University of Defence, Belgrade, Serbia; ^4^Department of Pharmacology and Toxicology, The Faculty of Medical Sciences, University of Kragujevac, Kragujevac, Serbia

**Keywords:** health economics, genetic polymorphisms, renal transplantation, pharmacogenetic tests, immunosuppressive drugs

## Abstract

Renal transplantation is the treatment of choice for the patients with end-stage renal failure. Genetic factors, among others, can influence variability in response to immunosuppressive drugs. Nowadays, due to restrictive health resources, the question arises whether routine pharmacogenetic analyses should be done in the renal transplant recipients or not. The aim of this literature review was to present the up-to-date information considering the economic feasibility of pharmacogenetic testing in patients subjected to renal transplantation. The organization United Network for Organ Sharing in the US estimated that total costs per renal transplant concerning these analyses were $334,300 in 2014. Pharmacogenetic testing prior to treatment initiation could be helpful to predict and assess treatment response and the risks for adverse drug reactions. This kind of testing before treatment initiation seems to be one of the most promising applications of pharmacokinetics. Although pharmacogenetic tests were found to be a cost-effective or cost-saving strategy in many cases, some authors represent another opinion. However, if the real costs of renal transplantation are recognized, the application of these tests in the standard daily practice could be considered more realistic, which additionally emphasizes the importance of future studies assessing their cost effectiveness.

## Introduction

End-stage renal disease is the last stage of the chronic renal disease. The most common causes of end-stage renal disease are diabetes and high blood pressure (1–3). In the general population, the prevalence of chronic renal disease varies approximately from 7 to 20% (4–8). Dialysis (hemodialysis and peritoneal dialysis) and renal transplantation are the treatments for the patients with end-stage renal failure. Renal transplantation is the treatment of choice for these patients since survival rate and quality of life are better in comparison to patients who are subjected to dialysis (9, 10).

According to US statistics, chronic kidney disease afflicted up to 20 million people, while more than 871,000 of patients were being treated for end-stage renal diseases in 2009 (11, 12). Also, 17,107 renal transplants were performed in 2014. Moreover, 100,791 people are currently estimated to be waiting for renal transplants (13). Pre-end-stage renal disease entails a cost in excess of $26,000 per patient per year (12). The World Health Organization, as the leading authority, is seriously concerned about these figures.

The major problems in renal transplantation are: the shortage of donors, adequate immunosuppression, allograft dysfunction and rejection, adverse reactions to drugs, therapeutic drug monitoring (TDM), restricted health resources, etc. However, the problems of greatest concern are the prevention of graft rejection and chronic toxicity of immunosuppresants due to their inadequate levels in the plasma. The inter- and intraindividual variability in response to immunosuppressive drugs is well known (14, 15). Gender, age, body mass index, hematocrit, diabetes status, liver dysfunction, drug interactions, etc., can also influence the variability in response to these drugs among patients (14, 16). In addition, genetic factors are likely to play a major role as well (17, 18).

Personalized medicine refers to the application of patient-specific profiles (incorporating genetic and genomic data) in order to assess individual risks and tailor prevention and disease-management strategies (19, 20). Therefore, a better control of immunosuppression should be achieved by pharmacogenetic testing (21).

Current recommended immunosuppression protocols in the patients subjected to renal transplantation include a combination of calcineurin inhibitors (tacrolimus or cyclosporine) and antiproliferative agents (mycophenolate mofetil or azathioprine), with and without regimens of corticosteroids (18, 22, 23). Tacrolimus as the first-line treatment is recommended by the Kidney Disease Improving Global Outcomes Transplant Work Group (KDIGO) (23). Mammalian target of rapamycin (mTOR) inhibitors (everolimus or sirolimus) should be used instead of calcineurin inhibitors but only after the graft function has been established and surgical wounds have healed (23). Induction therapy (interleukin 2 receptor antagonists) is used in high-immunological risk renal transplant recipients.

Gene polymorphism as a significant factor contributes to the variability in pharmacokinetics and pharmacodynamics of immunosuppressive drugs, which may result in their toxicity and/or lack of efficacy (14, 15, 24, 25). Therefore, pharmacogenetic analyses could be very important for immunosuppressive drug therapy. However, due to the restriction of health resources, the question arises whether renal transplantation management should include routine pharmacogenetic testing.

The aim of our study was to determine the economic feasibility of pharmacogenetic analyses in patients subjected to renal transplantation based on a review of relevant literature.

## The Impact of Genetic Polymorphisms on the Metabolism and Transport of Immunosuppressive Drugs

The TDM of immunosuppressive drugs helps the determination of suitable dose, and the trial and error approach to dosing is still a common everyday practice. Nowadays, the challenge is to combine pharmacogenetic with pharmacokinetic information in order to provide patients with the most suitable treatment.

Many pharmacogenetic studies have been conducted to evaluate the importance of genetic polymorphism for tacrolimus and cyclosporine therapy success, mostly in regard to CYP3A4/5 and P-glycoprotein (P-gp). The most prominent gene-dependent effect on tacrolimus pharmacokinetics has been observed for *CYP3A5* (26). CYP3A5 activity differs significantly among individuals, which is mainly due to its genetic polymorphism: those who have at least one wild-type allele (CYP3A5*1) are denoted as expressors, and homozygote carriers of non-functional alleles, such as CYP3A5*3, as non-expressors (27). Numerous studies on tacrolimus pharmacokinetics in transplant recipients consistently reported that carriers of CYP3A5*3/*3 genotype require significantly lower doses for both induction and the maintenance phase of the therapy (28–38). Similar effects of *CYP3A5* polymorphism on cyclosporine levels have been detected, but the reports remained largely inconsistent (29, 37, 39–41).

Although wide interindividual variations in CYP3A4 levels have been described (42), only few gene polymorphisms have been associated with altered *in vivo* enzyme activity (43, 44). In renal transplant patients, significantly lower daily tacrolimus dose requirements were observed in carriers of CYP3A4*18 and *22 alleles (41, 45–47), especially in CYP3A5 non-expressors (48–50). The same, but usually less, pronounced effect was detected in transplant recipients on cyclosporine therapy (40, 47, 48). On the other hand, CYP3A4*1B have been associated with higher tacrolimus dose requirements (51, 52), but the conclusions remained arguable, mainly due to the strong linkage disequilibrium between this *CYP3A4* allele and fully functional CYP3A5*1 (29).

P-glycoprotein is an efflux transporter involved in elimination and permeability restriction of numerous endogenous and xenobiotic compounds, including calcineurin inhibitors (53, 54). It is encoded by the highly polymorphic *ABCB1* (*MDR1*) gene, with more than 250 non-synonymous single nucleotide polymorphisms (SNPs) described so far (54). Of those, the most frequent and thus most extensively studied are 1236C > T, 2677G > T/A, and 3435C > T (53, 54). In the renal transplant recipients that do not express CYP3A5, 1236–2677–3435 TTT-TTT diplotype was highly associated with lower tacrolimus dose requirements (38), and similar effect was observed when individual *ABCB1* polymorphisms were considered (55–57). In patients on cyclosporine therapy, significantly higher daily dose requirements were found in 2677GG and 3435CC genotype carriers (58, 59). However, there are studies that failed to find any significant association between pharmacogenetics of P-gp and pharmacokinetics of calcineurin inhibitors (60–62); therefore, the proposed relationship is still considered controversial.

Inhibitors of mTOR are substrates for CYP3A4/5 and CYP2C8 enzymes and P-gp (63). However, pharmacogenetic studies in regard to sirolimus and everolimus treatment are scarce and with conflicting results. In renal transplant recipients, significantly higher sirolimus dose requirements were observed in carriers of CYP3A4*1B allele or in the absence of CYP3A5*3, while *ABCB1* polymorphism did not affect drug pharmacokinetics (64–66). The effect of CYP3A5*3 on everolimus was investigated, but not detected (67, 68).

Based on a considerable body of evidence in favor of clinically relevant genotype–phenotype association, a guideline for *CYP3A5* genotype and tacrolimus dosing was published (69). In regard to other calcineurin and mTOR inhibitors and their disposition-related genes, there are currently not enough data to support routine pharmacogenetic testing (70).

Azathioprine is a precursor of 6-mercaptopurine (6-MP), further metabolized to active thioguanine nucleotide (TGN) metabolites through a multi-step process (71, 72). Glutathione *S*-transferase has been implicated in the formation of 6-MP, which is then inactivated in the liver by highly polymorphic thiopurine *S*-methyltransferase (TPMT), as well as by xanthine oxidase (73). Heterozygous and homozigous carriers of non-functional *TPMT* alleles (mainly *2, *3A, *3B, *3C, and *4) have low and deficient enzyme activity, respectively, and are at significantly higher risk of adverse drug reactions to azathioprine (73–76). Thus, the routine genotyping for *TPMT* polymorphisms prior to the initiation of the therapy has become a cornerstone of thiopurines-based treatment (77).

Mycophenolat mofetil is another prodrug, which requires enzymatic hydrolysis for activation (78). Mycophenolic acid then undergoes further biotransformation, which includes glucuronidation as the major metabolic pathway. Several uridine 5′-diphospho glucuronosyl transferases (UGTs) are involved in the process. However, UGT1A9 is of special importance, as certain SNPs of its coding gene, as well as of the genes coding for drug transporters (such as SLCO1B1), lead to a significantly lower drug exposure and a higher risk of acute transplant rejection (79, 80). In addition, the gene coding for inosine monophosphate dehydrogenase (IMPDH), the target of mycophenolic acid, is also polymorphic, and the association of certain *IMPDH* SNPs with the immunosuppressive response were reported (81). However, the data on the impact of genetics on the drug efficacy and safety are still conflicting (70, 78), Therefore, currently, there are no recommendations for routine pharmacogenetic testing in regard to mycophenolat mofetil therapy.

In regard to the use of biologic agents and corticosteroids in induction and maintenance therapy, no evidence showing the association between genetic polymorphisms and their pharmacokinetics or pharmacodynamics was found in the literature (82).

## The Economic Evaluation of Renal Transplantation

The history of health economics in recent decades (83) teaches us that the burden of non-communicable “prosperity” illnesses is among the top causes of growing costs of health care (84). This landscape of medical care spending has evolved considerably across the Globe with developing and emerging economies taking ever larger share of global expenditure on health (85). This fact is taking its toll since the Third World nations are facing double burden: the old unresolved pool of infectious diseases and another rapidly expanding one attributable to the non-communicable diseases (NCD) (86). Coping with the challenges outsourcing from cancer, diabetes, fertility issues, mental disorders, and ultimately renal failure, thus, is made substantially more difficult. These systems were traditionally shaped to combat acute communicable diseases and injuries at the first place (87). Now they face chronic illnesses, which are far more demanding in terms of social capital and medical resources that need to be devoted (88). Among the few bright success stories in lessening the grip of major NCDs are certainly the BRICS nations (89). Such change of morbidity has clearly reflected on the demand for pharmaceuticals and national spending on medicines in a large number of countries (90, 91). In case of immunosuppressants used in renal transplantation procedures, this growth was significant both in prescription and value-based terms (92). Surgical services, imaging diagnostics, and home-based care, next to dialysis, should not be forgotten as major cost drivers in terminal renal failure treatment.

Nowadays, a renal transplantation is considered the treatment of choice for many people with severe chronic kidney disease, but there is a shortage of organs available for donation. Many people who are candidates for kidney transplantation are just put on a transplant list to wait for an available organ. However, the waiting time on the list may vary considerably.

The costs of renal transplantation include transplant evaluation and testing, transplant surgery, follow-up care, and medication. After a renal transplantation, patients will need several drugs, including immunosuppressants, to sustain the transplanted kidney. Costs per one transplant patient in Serbia for a 10-year period were calculated to be €48,949 (93). On the other hand, according to the US United Network for Organ Sharing (UNOS), the first year billed charges for a kidney transplant are more than $262,000 (94). The total monthly costs of Cellcept^®^, Prograf^®^, Prednisone^®^, and Myfortic^®^ are approximately $1,064, $1,340, $12, and $806, respectively. The 2-year costs of four different immunosuppressive strategies (sirolimus, everolimus, cyclosporine, or tacrolimus) have been shown to vary between €26,732 and €49,978 (95). Therefore, in the time of restricted health resources, the choice of therapy is very important.

Renal transplantation costs differ significantly worldwide. For example, the total cost of renal transplantation procedure in Iran was $9,224 (96). The immunosuppressive therapy accounted for 65.8% ($6,076), only in the first year. The transplantation procedure costs were $2,048, while organ procurement was about $1,100. On the other hand, the total costs of the first year after transplantation in Sudan were US$14,825 and after that approximately US$10,651 (97). The renal transplant cost estimate in India was about US$70,000 for laparoscopic kidney transplantation, while open nephrectomy costs were US$100,000 (98). In the US, laparoscopic kidney transplantation and open nephrectomy cost approximately US$300,000 and US$450,000, respectively.

**Figure 1:**
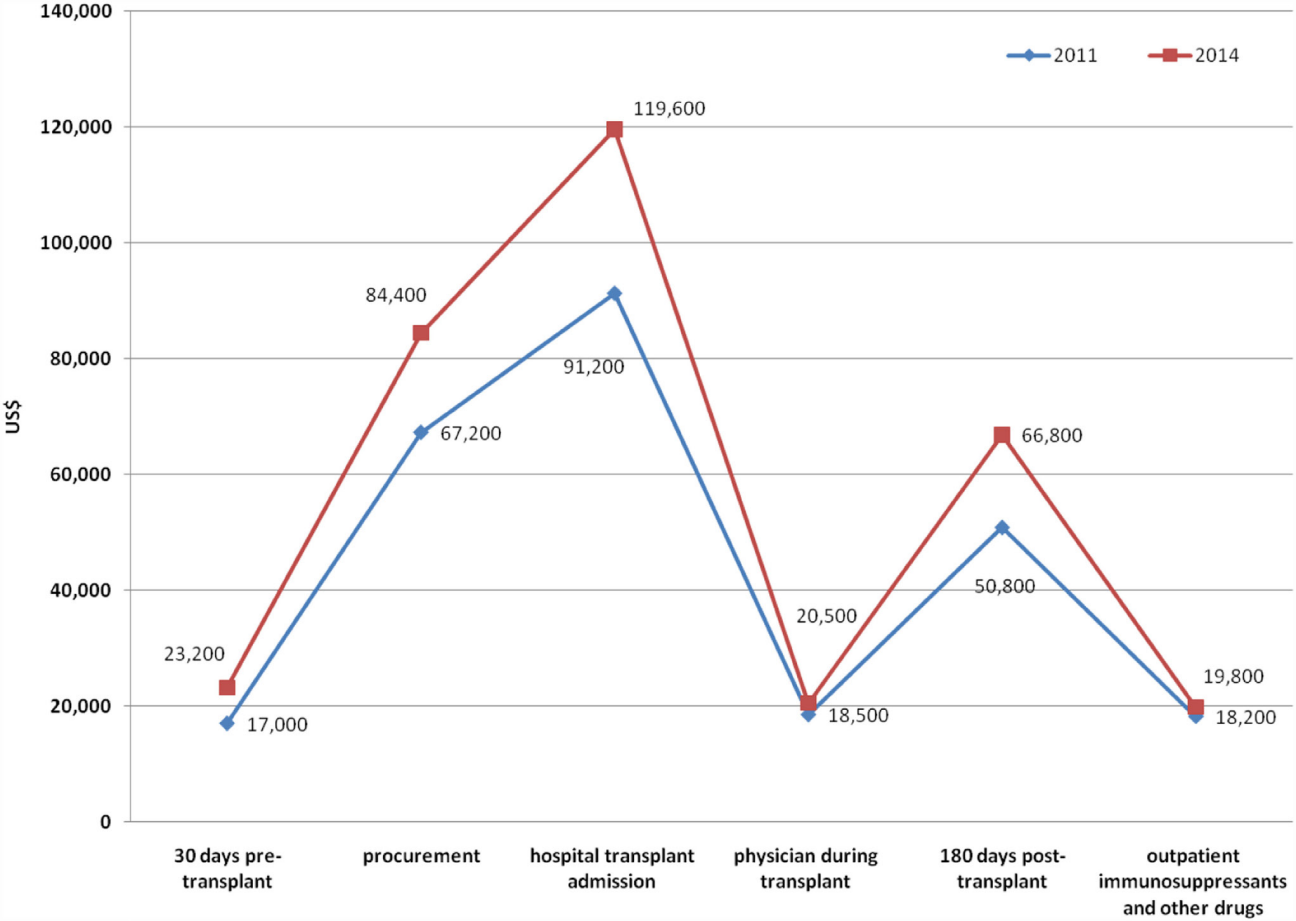
**The costs of renal transplantation in 2011 and 2014 according to the US United Network for Organ Sharing (UNOS)**.

Cost estimates of UNOS per renal transplant concerning 30 days pre-transplantation, procurement, hospital transplant admission, physician services during transplant, 180-days posttransplant, and administration of immunosuppressants in 2011 and 2014 in the US are represented in Figure 1 (99). The total costs were $262,900 and $334,300 in 2011 and 2014, respectively (99–101).

## The Economic Evaluation of Pharmacogenetic Tests

Pharmacogenetic testing prior to the treatment initiation could be helpful to assess both the risks of adverse drug reactions and treatment response (21, 102). Moreover, pharmacogenetics-based testing before treatment initiation seems to be one of the most promising applications of drug pharmacokinetics.

The cost effectiveness of genotyping prior to the therapy with some drugs, such as abacavir, allopurinol, carbamazepine, clopidogrel, and irinotecan, indicates that genotyping was justified when it was performed on a large numbers of patients. However, evaluation of cost effectiveness of genotyping prior to the use of immunosuppressive drugs has not been performed yet.

Provenzani et al. concluded that, considering the relatively high costs of pharmacogenetic tests and availability of TDM, the genotyping of all transplant patients is not affordable in many countries (103). This situation may change in the near future since studies on pharmacogenetics would produce valuable data, and the improvements in the genotyping analyses will decrease the costs associated with this type of tests. However, prospective clinical studies must show that genotype determination before transplantation allows for the better use of given drugs and improves their safety and clinical efficacy. Currently, genetic tests determining a patient’s CYP2D6 and CYP2C19 polymorphisms cost from $350 to $400, not including the markup and some other costs associated with the test. Moreover, most genetic tests cost a few hundred dollars at the moment, but they are estimated to become less expensive in the future (104–106). However, to justify the costs of genetic testing, genotypic analyses have to demonstrate their ability to significantly improve transplant patient outcomes and show cost savings.

The pharmacogenetic tests prior to the treatment with azathioprine represent a good example of saving money. The cost-effectiveness model for azathioprine, based on parameters collected for TPMT genotyping costs, estimates for frequency of TMPT deficiency, rates of thiopurine-mediated myelosuppression in TPMT-deficient individuals, and myelosuppression-related hospitalization costs, established that TPMT testing in all patients had a favorable cost-effectiveness ratio (107). The costs of a genotype test were estimated on about $NZ30 (€18.90) (108). In another study, it was estimated that costs of the TPMT screening (genotype) were $510.06 (109). These authors concluded that the least costly treatment strategy was TPMT screening, with 1-year costs saving per patient of $3,281. The mean calculated cost per life year, gained by TPMT genotyping in all patients in the four studied countries (Germany, Ireland, Netherlands, and the United Kingdom), was €2,100, based on genotyping costs of €150 per patient (107).

Thervet et al. have recommended a successful protocol of tacrolimus starting dose determined by patient’s genotype: CYP3A5*3*3 are to be given 0.15 mg/kg/day, while CYP3A5*1*1 or *1*3 expressers should be treated with 0.30 mg/kg/day (110). On the base of this protocol, in the group receiving the adapted dose, a higher proportion of patients had values within the targeted *C*_0_ on day 3 after the initiation of tacrolimus. Namely, they required fewer dose modifications while the targeted *C*_0_ was achieved by 75% of these patients more rapidly. This pharmacogenetic test prior to tacrolimus initiation could be helpful to reduce the risks of adverse drug reactions due to the patient overexposure, as well as to reduce the costs due to the faster achievement of the optimal tacrolimus concentrations.

The substantial increase of health expenditure, due to the rapid population growth and aging became a great concern for governments all over the world (111, 112). On the one hand, while extensive literature on the determinants of health expenditure in the Organization for economic co-operation and development (OECD) countries can be found, that is not the case with the situation in developing countries (101). Literature data show great variations between countries concerning health expenditure, since gross domestic product share ranges from 5 to 15%. For example, health expenditure per capita of US$9,715 in Norway is the biggest, while US$13 in the Central African Republic is the smallest.

There are many factors that account for the enormous costs of pharmacogenetic analyses. Devices themselves, with the current price of about $10,000, together with trained staff necessary to perform analyses, are the most expensive ones. It is obvious that these costs are high even for the developed countries, whereas for the developing countries, they are unaffordable for the time being (113).

## Conclusion

Due to the lack of appropriate cost-effectiveness studies, and the availability of TDM analyses, from an economic point of view, we could neither recommend nor discourage the use of pharmacogenetic tests as a routine clinical practice in the everyday treatment of renal transplant patients. Future recommendations will depend on robust clinical evidence regarding pharmacogenetic test efficacy and precise costs concerning renal transplantation.

## Author Contributions

NR coordinated the teamwork of coauthors, wrote the paper, and searched for literature. VD-S assisted with the writing of the article, searched for literature, and had done the critical revision of the text. NV assisted with the writing of the article and searched for literature. AK assisted with the writing of the article and searched for literature. ZS assisted with the writing of the article and searched for literature. ND assisted with the writing of the article, searched for literature, and had done the critical revision of the text. All authors read and approved the final manuscript.

## Conflict of Interest Statement

The authors declare that the research was conducted in the absence of any commercial or financial relationships that could be construed as a potential conflict of interest.
